# Microcystin-LR Induced Reactive Oxygen Species Mediate Cytoskeletal Disruption and Apoptosis of Hepatocytes in *Cyprinus carpio* L.

**DOI:** 10.1371/journal.pone.0084768

**Published:** 2013-12-20

**Authors:** Jinlin Jiang, Zhengjun Shan, Weili Xu, Xiaorong Wang, Junying Zhou, Deyang Kong, Jing Xu

**Affiliations:** 1 Nanjing Institute of Environmental Sciences/Key Laboratory of Pesticide Environmental Assessment and Pollution Control, Ministry of Environmental Protection, Nanjing, People’s Republic of China; 2 State Key Laboratory of Pollution Control and Resource Reuse, School of the Environment, Nanjing University, Nanjing, People’s Republic of China; University of Navarra School of Medicine and Center for Applied Medical Research (CIMA), Spain

## Abstract

Microcystins (MCs) are a group of cyclic hepatotoxic peptides produced by cyanobacteria. Microcystin-LR (MC-LR) contains Leucine (L) and Arginine (R) in the variable positions, and is one of the most common and potently toxic peptides. MC-LR can inhibit protein phosphatase type 1 and type 2A (PP1 and PP2A) activities and induce excessive production of reactive oxygen species (ROS). The underlying mechanism of the inhibition of PP1 and PP2A has been extensively studied. The over-production of ROS is considered to be another main mechanism behind MC-LR toxicity; however, the detailed toxicological mechanism involved in over-production of ROS in carp (*Cyprinus carpio* L.) remains largely unclear. In our present study, the hydroxyl radical (•OH) was significantly induced in the liver of carp after a relatively short-term exposure to MC-LR. The elevated reactive oxygen species (ROS) production may play an important role in the disruption of microtubule structure. Pre-injection of the antioxidant N-acetyl-cysteine (NAC) provided significant protection to the cytoskeleton, however buthionine sulfoximine (BSO) exacerbated cytoskeletal destruction. In addition, the elevated ROS formation induced the expression of apoptosis-related genes, including p38, JNKa, and bcl-2. A significant increase in apoptotic cells was observed at 12 - 48 hours. Our study further supports evidence that ROS are involved in MC-LR induced damage to liver cells in carp, and indicates the need for further study of the molecular mechanisms behind MC-LR toxicity.

## Introduction

Microcystins (MCs) are a group of cyanobacterial toxins comprised of more than 80 variants. MC-LR is both one of the most common variants and one of the most potently toxic peptides, containing amino acids Leucine (L) and Arginine (R) in the variable positions. The outbreak of a cyanobacterial bloom induces the release of MCs into water and represents a serious threat to aquatic ecosystems [1]. Previous studies have shown that the death of large numbers of fish during outbreaks of cyanobacterial bloom is associated with the production of MCs and with several special conditions, including high water temperature, high pH, high concentration of ammonia and nitrogen, and low dissolved oxygen [2]. It is well known that MCs can cause a variety of toxic effects in fish through various pathways. MC exposure can cause histopathological changes in various organs, including the liver, kidney, gill, intestine, and heart. It can also alter the activity of various enzymes in the fish. In addition, MC exposure can affect growth rate, osmotic pressure, heart rate and behavior [1,3]. Many laboratory and field studies have demonstrated that MCs can accumulate in various tissues and organs of fish (mainly in the liver, but they can also be detected in muscle tissues). The long-term accumulation of toxins in fish will definitely produce harmful effects, and may also affect human health through the food chain [4-9].

A number of exposure routes have been used to study the effects of MCs on fish, including intraperitoneal injection, feeding, immersion in water containing purified toxins (MC-LR or MC-RR), cyanobacteria crude extract, and whole cells of cyanobacteria. Intraperitoneal injection is the most commonly used technique, due to the rapid onset of toxicity. The mechanism behind the toxicity of MCs in fish is similar to that in mammals, causing irreversible inhibition of protein phosphatases PP1 and PP2A in fish liver cells [10,11]. This leads to excessive phosphorylation of proteins, alterations in the cytoskeleton, loss of cell shape and subsequent destruction of liver cells, causing hepatic hemorrhage or hepatic insufficiency [12]. MCs are also responsible for an increase in oxidative stress, which can subsequently trigger apoptosis [13]. However, the association between intracellular ROS levels and other toxicities in fish remains unclear. 

The cytoskeleton consists of three major structural elements: microtubules, microfilaments, and intermediate filaments. These elements play an important role in maintaining cellular architecture and internal organization, cell shape, motility, cell division, and many other processes [14]. It has been reported that microtubules can be disrupted by cyanobacteria extract or purified MCs in primary cultured rat hepatocytes and several non-hepatocyte cell lines [14,15]. Ding et al. [14] suggested that intracellular GSH plays an important role in MC-induced cytotoxicity and cytoskeleton changes in primary cultured rat hepatocytes. However, the role of the excessive production of ROS in this biological process has not been fully elucidated. In addition, it would be interesting to ascertain whether and how MC-LR could induce similar effects on the cytoskeleton system in fish liver cells, a question which has received little attention so far.

Carp (*Cyprinus carpio* L.), a common fish that are widely distributed in Asia, including China, were chosen to study the toxic effects of MC-LR. In our present study, the effects of sublethal doses of MC-LR on the ROS level, HSP70 expression, cytoskeletal structure, and apoptosis in liver cells were investigated. The results obtained in this study help to reveal the association between intracellular ROS and other toxic effects induced by MC-LR and to further examine the detailed toxicological mechanisms behind MC-LR-induced toxicity.

## Materials and Methods

### Ethics Statement

This study was approved by the Animal Ethics Committee of the Nanjing Institute of Environmental Sciences, Ministry of Environmental Protection. The institute does not issue a number or ID to any animal study, but the ethical committee approved and helped guide the animal use in this study.

### Chemicals and reagents

MC-LR (purity > 96%) was purchased from Alexis Biochemicals (Läufelfingen, Switzerland). N-tert-Butyl-a-phenylnitrone (PBN, purity > 98%) was purchased from J&K Chemical (USA). Paraffin, hematoxylin, eosin, glutaraldehyde, uranyl acetate and lead citrate were purchased from Sigma Chemical (St. Louis, MO, USA). Dimethyl sulfoxide (DMSO) and methanol were purchased from Tedia (Fairfield, OH, USA). Other reagents were analytical grade and obtained from chemical companies in China.

### Fish and experimental designs

Six month old carp, with an average body length of 14.00 ± 1.08 cm and weight of 29.26 ± 5.09 g, were obtained from a pilot research station of the Freshwater Fisheries Research Center (FFRC), Chinese Academy of Fishery Sciences. These fish were acclimated to laboratory conditions for 14 days with dechlorinated tap water. Fish were fed commercial pellet food daily during the acclimation and test periods, except for the last two days of acclimation. During experiments, water temperature was 16.1 ± 0.2 °C, pH value was 7.20 ± 0.35, dissolved oxygen was 8.6 ± 0.5 mg/L, the photoperiod was 12 h/12 h, and total hardness was 129.7 ± 8.3 mg as CaCO_3_ per liter. Water was constantly aerated during the acclimatization and test periods.

Carp were randomly divided into three groups with 40 carp per group. Each group was treated with either 50 μg/kg of MC-LR, 120 μg/kg of MC-LR, or saline, by intraperitoneal injection (MC-LR was dissolved in saline). An equal volume of saline was administrated and used as a control. Each group was then subdivided into five groups with 8 fish per group and carp were sacrificed at 1, 5, 12, 24 and 48 hours after exposure to MC-LR. Livers were quickly taken out for immediate use or frozen in liquid nitrogen before storage at a temperature of -80°C for further analysis. 

### ROS trapping and EPR measurement

ROS levels were determined by electron paramagnetic resonance (EPR), according to the method described by Luo et al. [16] and Jiang et al. [17]. About 0.1 g of liver tissues were quickly homogenized with a cold glass homogenizer in 1.0 ml of 50 mM PBN dissolved in DMSO. The homogenates were transferred to quartz capillary tubes and then immediately stored in liquid nitrogen for EPR analysis. All operations were performed in a sealed box that was purged continuously with nitrogen gas. The EPR spectra were recorded with a Bruker EMX 10/12 X-band spectrometer (Bruker, Germany) under the same conditions described by Luo et al. [16] and Jiang et al. [17]. The height of the second peak of the EPR signals was interpreted as the intensity of •OH in liver tissues. All experiments were performed in quadruplicate.

### Immunohistochemistry

Immunohistochemistry was performed according to the method described by Jiang et al. [17] using Rabbit Anti-HSP70 (Fish) Polyclonal Antibody (Stressgen, USA) at a dilution of 1:800. Sections (4 to 5 μm) were mounted on silane-coated slides and stained with the SP-9001Histostain™-Plus Kit (Zymed, USA) according to the manufacturer's instructions. The mean integrated optical density (MIOD) of the HSP70 expression-positive area was calculated using Image-Pro Plus software. At least six fields were calculated per slide.

### Laser scanning confocal microscopy

Cytoskeletal changes were evaluated based on the expression and distribution of beta-tubulin. Double fluorescence immunohistochemistry sections of 5 μm were cut from paraffin blocks and mounted on gelatin/chrome alum-coated glass slides. The paraffin sections were deparaffinized in xylene and rehydrated in graded ethanol and distilled water. The non-specific binding sites were blocked in 5% normal goat serum diluted in 1X PBS with 0.3% Triton X-100 for one hour at room temperature. Beta-tubulin was stained overnight with an Alexa Fluor® 488-conjugated monoclonal antibody against beta-tubulin (9F3, Sigma) at a 1:500 dilution at 4°C. The antibody was diluted in 1X PBS containing 1% BSA and 0.3% Triton X-100. DAPI (4’,6-diamidino-2-phenylindole) was used to stain the nucleus. The sections were measured using a Zeiss LSM 710 laser scanning confocal microscope (Zeiss, Oberkochen, Germany). The green fluorescence of Fluor® 488 was excited at 488 nm and the blue fluorescence of DAPI was excited at 405 nm.

### Real time quantitative PCR

Total RNA was isolated from the liver samples using the TRIzol reagent (Invitrogen) according to the manufacturer’s instructions, and treated with DNAase to eliminate residual DNA prior to reverse transcription of total RNA to complementary DNA (cDNA). The concentration of total RNA was measured by absorbance at 260 nm. The purity was estimated by the 260/280 nm absorbance ratio. One microgram of total RNA was characterized by denaturing agarose gel electrophoresis. Primers for the evaluation of bcl-2 (forward primer: 5’-TTTCGCTCAGAAGTGACGGC-3’; reverse primer: 5’-GCAGTGCGGTGCTGAAAGAT-3’), JNKa (forward primer: 5’-TAAAACACCTCCACTCGGCG-3’; reverse primer: 5’-GCCAGACCGAAATCCAGGA-3’), and p38 (forward primer: 5’-TGGAAACGGCTCACGTATGAA-3’; reverse primer: 5’- TCTGGATGAAGGTCCTGGAGG-3’) gene expression were designed using Primer Express 2.0 (Applied Biosystems). The cDNA was synthesized from 0.5 μg of total RNA, using the PrimerScript RT reagent Kit (TaKaRa) on a GeneAmp® PCR System 9700 (Applied Biosystems) according to the manufacturer’s protocol.

Quantification of gene expression was carried out on a 7500 Fast Real-Time PCR System (Applied Biosystems). The reaction mixture was composed of 10 μl of SYBR Green RT-PCR Master Mix (TaKaRa), forward and reverse primers (10 μM, 0.4 μl each), ROX Reference Dye II (50×, 0.4 μl), 7.8 μl of nuclease-free water, and the cDNA sample (1 μl). The PCR amplification protocol was 95°C for 15 seconds followed by 40 cycles of 95°C for 5 seconds and 62°C for 34 seconds. After PCR, a melting curve analysis was performed to demonstrate the specificity of the PCR product, which was displayed as a single peak. Every sample was analyzed in quadruplicate. Differences in expression levels were calculated using the 2^-∆∆Ct^ method [18]. Amplification of 18S rRNA (Applied Biosystems) was used as an internal control for bcl-2, JNKa and p38 expression [19]. Statistical significance was determined using a one-way ANOVA, followed by Duncan’s multiple range test (SPSS, Inc., Chicago, IL). Data are presented as means with standard errors (mean ± SE). A *p*-value < 0.05 was considered statistically significant. 

### Flow cytometry

Cells were isolated from fresh carp liver. Briefly, the livers were cut into pieces and put into a cell separator containing 0.1 M phosphate buffer (pH = 7.4) and chopped for 1 min (the Becton Dickinson Medimachine, USA). The cells were then filtered through a membrane (STERIKING 75 mm, WIPAK Medical, Finland) and then centrifuged at 1000 rpm for 5 min. Cells were collected, washed, centrifuged and resuspended in 1% (w/v) paraformaldehyde in PBS (pH 7.4). They were then kept on ice for 1 hour, washed in PBS, centrifuged, and fixed in 70% (v/v) ice-cold ethanol at -20 °C for 12 hours, before being stained with terminal deoxynucleotidyl transferase (TdT) and FITC-labeled deoxyuridine triphosphates (FITC-dUTP). A commercial TUNEL kit (APO-DIRFCT^TM^ Kit, BD Biosciences) was used to perform the TUNEL staining of liver cells according to the manufacturer’s instructions. TUNEL is a method for detecting the 3'-OH ends of DNA exposed during the internucleosomal cleavage that occurs during apoptosis. TUNEL assays identify apoptotic cells by TdT-mediated addition of labeled (X) deoxyuridine triphosphate nucleotides (X-dUTPs) to the 3’-OH end of DNA strand breaks, which are subsequently visualized depending on the introduced label. Apoptosis of liver cells was analyzed using a FACSCalibur flow cytometer (BD Biosciences).

### Statistical analysis

 Data were expressed as mean ± standard error (SE) and analyzed using a one-way ANOVA. Significant differences between means were determined with the LSD-*t* test. Differences were considered to be significant at *p* < 0.05 (*) and *p* < 0.01 (**).

## Results

### Effects of MC-LR on hepatic ROS levels in carp liver

**Figure 1:**
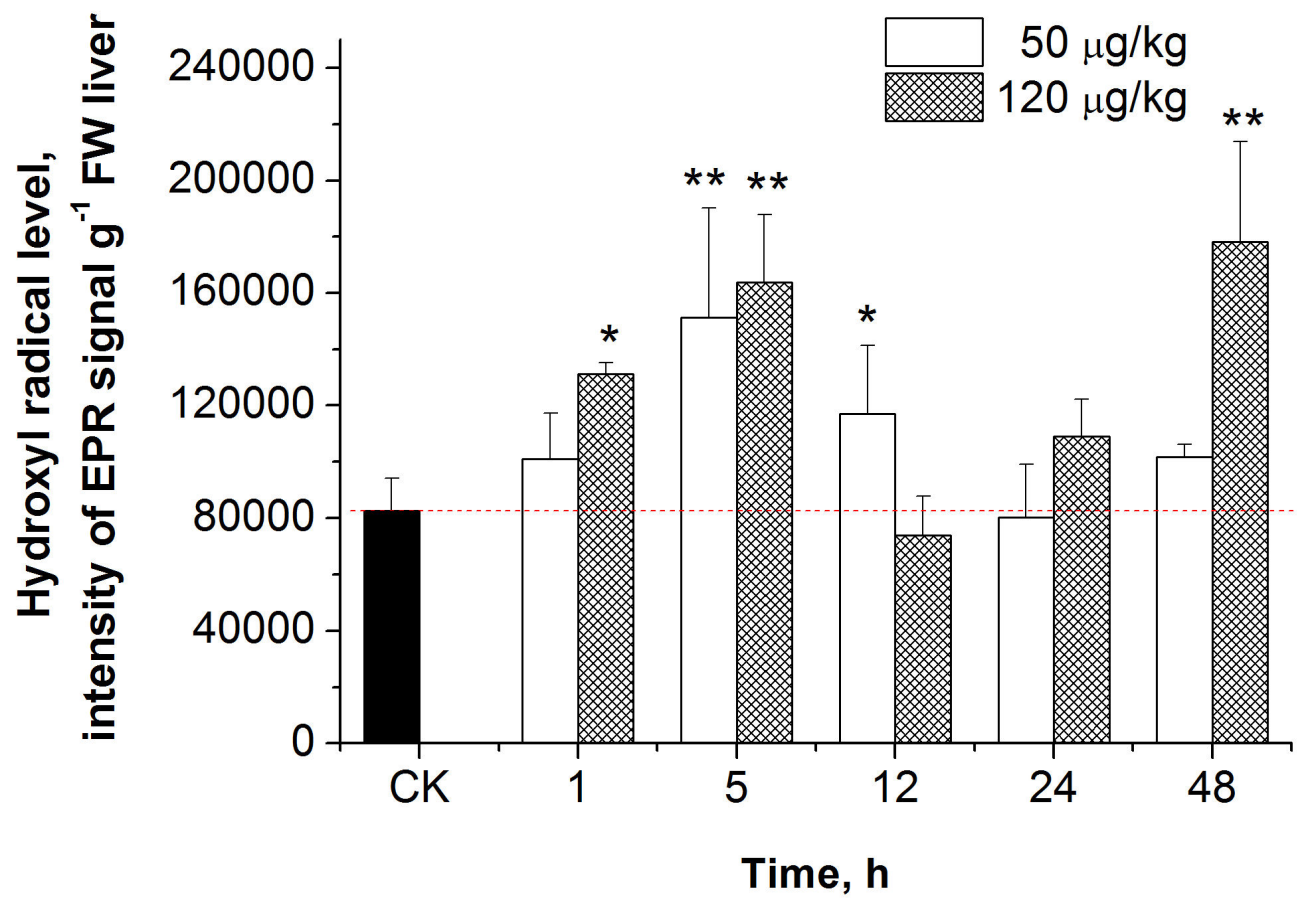
Effect of sublethal doses of MC-LR on •OH production in the liver of *C. carpio*. Data are presented as mean ± SE (n = 4). CK stands for control group. Asterisk indicates the statistical difference relative to the control.

Typical EPR spectra of the carp hepatic ROS signal, induced by MC-LR, consisted of three groups with two hyperfine splitting peaks in each, with identical intensity. These six line spectra were identified as radical hydroxyls (•OH) with hyperfine splitting constants of g = 2.0057, a^N^ = 13.88 G, a^H^ = 2.35 G [20]. The intensity of •OH in different groups is shown in Figure 1. The intensity of •OH in the group treated with 50 μg/kg of MC-LR was significantly increased at 5h and 12 h after MC-LR exposure, compared to the control group (*p* < 0.05, *p* < 0.01). The intensity of •OH in the group treated with 120 μg/kg of MC-LR was significantly increased at 1, 5, and 48 hours after MC-LR exposure, compared to the control group (*p* < 0.05, *p* <0.01, *p* < 0.01). Of note, signal intensity of •OH was also seen in the control group, indicating that •OH can also be produced under normal physiological conditions.

### Effects of MC-LR on HSP70 in carp liver

**Figure 2:**
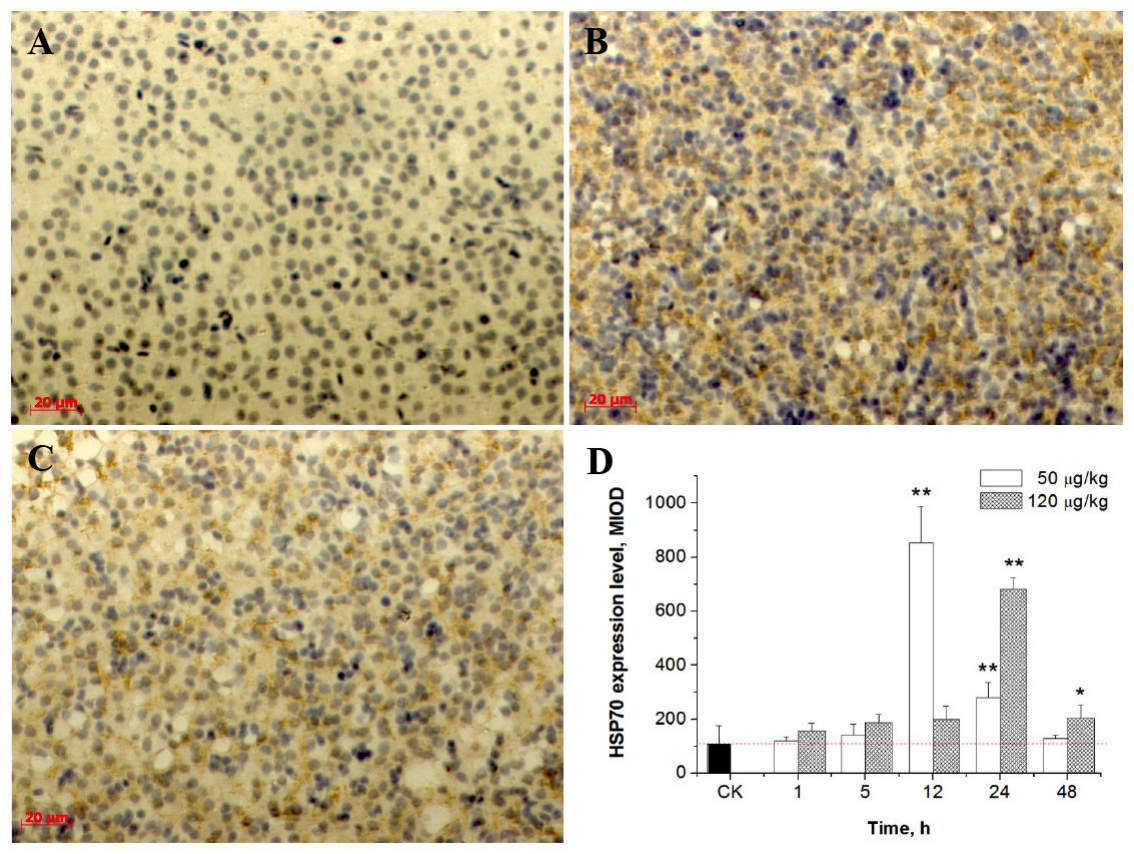
Effect of sublethal doses of MC-LR on HSP70 in the liver of *C. carpio*. (A) CK (control group); (B) 50 μg/kg MC-LR treated group at 12 h; (C) 120 μg/kg MC-LR treated group at 12 h; (D) Relative expression of HSP70 in the liver of *C. carpio* after intraperitoneal injection with MC-LR at 50 μg/kg and 120 μg/kg. Asterisk indicates the statistical difference relative to the control.

Expression of HSP70 in the liver was measured using immunohistochemical methods. As shown in Figure 2A-C, the obvious yellow stain indicates the presence of HSP70. Image-Pro Plus software was used to calculate the average cumulative optical density (MIOD) based on HSP70 staining. The control group exhibited less HSP70 protein expression. As shown in Figure 2D, a 7.09 fold increase in HSP70 was observed in the group treated with 50 μg/kg of MC-LR after a 12 h exposure, as compared to the control group. A significant increase in HSP70 expression was observed in the 50 μg/kg group after 12 h and 24 h exposures to MC-LR, compared to the control group (*p* < 0.01). A significant increase in HSP70 expression was observed in the 120 μg/kg treated group after 24 h and 48 h exposures to MC-LR, compared to the control group (*p* < 0.05, *p* < 0.01). A 6.27 fold increase in HSP70 was observed in the 120 μg/kg treated group after a 24 h exposure to MC-LR, as compared to the control group.

### Effects of MC-LR on the cytoskeleton in carp liver cells

**Figure 3:**
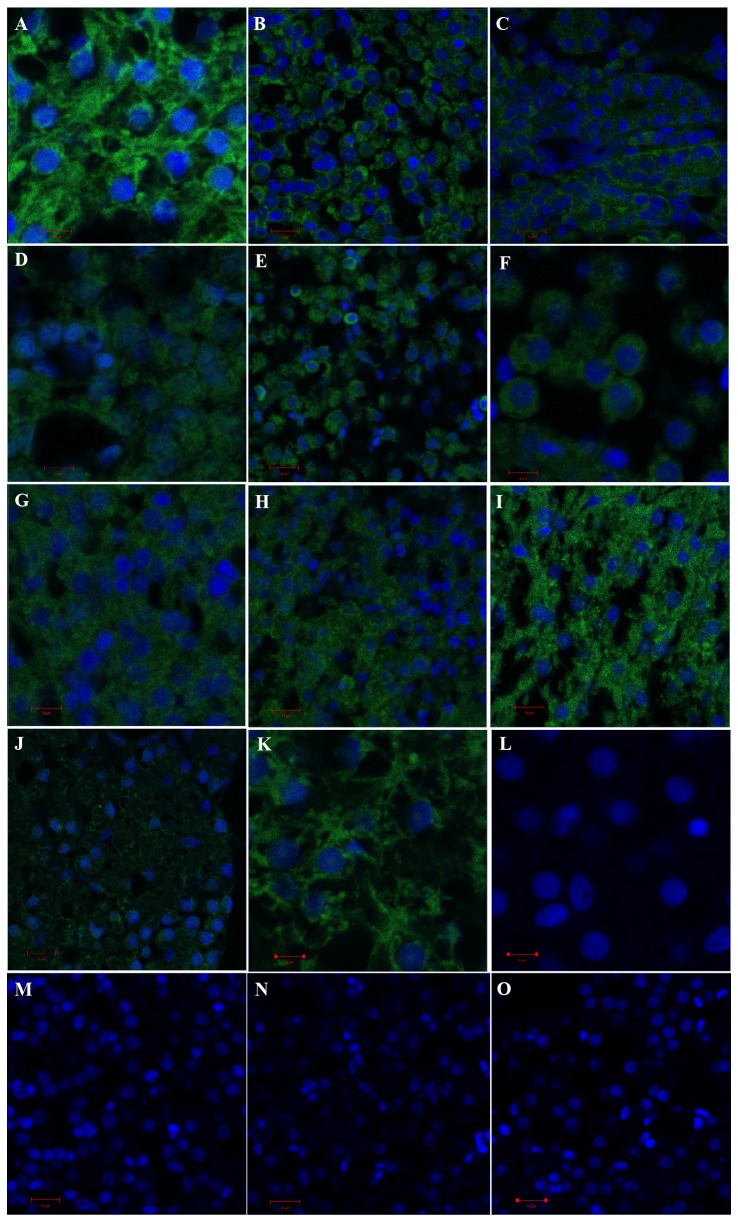
Effect of sublethal doses of MC-LR on the cytoskeletal system in the liver of *C. carpio*. (A) Control group, (scale bar = 5 µm); (B) 50 μg/kg MC-LR treated group, 12h, (scale bar = 10 µm); (C) 50 μg/kg MC-LR treated group, 48 h, (scale bar = 10 µm); (D) 120 μg/kg MC-LR treated group, 1 h, (scale bar = 5 µm); (E) 120 μg/kg MC-LR treated group, 5 h, (scale bar = 10 µm); (F) 120 μg/kg MC-LR treated group, 12 h, (scale bar = 5 µm); (G) 120 μg/kg MC-LR treated group, 24 h, (scale bar = 5 µm); (H) 120 μg/kg MC-LR treated group, 48 h, (scale bar = 10 µm); (I) pre-injection of 200 mg/kg of NAC 1 h before injection of 120 μg /kg MC-LR, 48 h, (scale bar = 10 µm); (J) pre-injection of 200 mg/kg of BSO 1 h before injection of 50 μg /kg MC-LR, 48 h, (scale bar = 10 µm); (K) intraperitoneal injection of 200 mg/kg of BSO for 48 h, (scale bar = 5 µm); (L) Nucleus of control group, (scale bar = 5 µm); (M) Nucleus of 50 μg/kg MC-LR treated group, 12 h, (scale bar = 10 µm); (N) Nucleus of 120 μg/kg MC-LR treated group, 5 h, (scale bar = 10 µm); (O) Nucleus of 120 μg/kg MC-LR treated group, 12 h, (scale bar = 10 µm).

Immunofluorescence staining of beta-tubulin was used to evaluate changes in the cytoskeleton. Figure 3 shows the morphologies of hepatic cells stained for microtubules and nuclei. Cytoskeletal changes were dependent on the dose and the exposure time of MC-LR. In the control group, the nuclei were widely surrounded by highly organized microtubules with normal morphology (Figure 3A), whereas in the 50 μg/kg treated group, cytoskeletal proteins were condensed around the nucleus, as evident after a 12 h exposure to MC-LR (Figure 3B). Moreover, liver cells had a hollow nucleus with condensed chromatin and exhibited apoptotic properties (Figure 3M) as compared to the control group (Figure 3L). After a 48 h exposure to 50 μg/kg of MC-LR, the cytoskeletal structure of most liver cells returned to normal (Figure 3C). Similarly, the condensed cytoskeletal proteins could be clearly observed around the nucleus in the 120 μg/kg treated group after 5 h and 12 h exposures to MC-LR (Figure 3E, 3F). The liver cells present in these two groups had more obvious hyperchromatic nuclei and nuclear pyknosis, with more irregular and hollow nuclei (Figure 3N, 3O). After a 24 h exposure to 120 μg/kg of MC-LR, the cytoskeletal structure had been restored (Figure 3G), however, the cytoskeletal protein content in the 120 μg/kg treated group was still lower when compared to the control group at 48 h (Figure 3H). 

In addition, the effects of 200 mg/kg of N-acetylcysteine ​​(NAC, a GSH synthesis precursor) and buthionine sulfoximine (BSO, a GSH synthesis inhibitor) on the cytoskeleton were studied. As shown in Figure 3H and 3I, NAC pretreatment had a significant protective effect on cytoskeletal proteins, as early as 1 h after exposure to MC-LR. BSO treatment (Figure 3K) induced hepatocellular nucleus condensation and decreased the skeletal protein content – a less serious effect than that caused by BSO + MC-LR treatment. Figure 3J shows that the cytoskeletal structure of the liver cells had been destroyed, and a considerable number of nuclei had disappeared at 48 h in the 200 mg/kg BSO + 50 μg/kg MC-LR treated group.

### Effects of MC-LR on the expression of mRNA in carp liver

**Figure 4:**
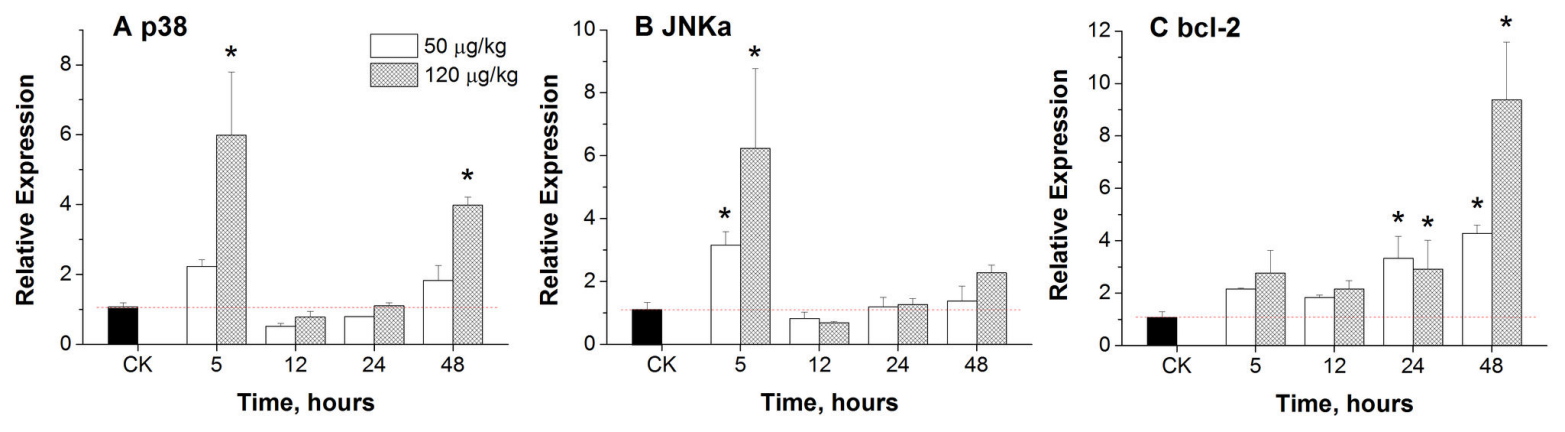
Effect of sublethal doses of MC-LR on mRNA expression in the liver of *C. carpio*. (A) p38; (B) JNKa; (C) bcl-2. Data are presented as mean ± SE (n = 4). Asterisk indicates the statistical difference relative to the control.

Genes involved in apoptosis, such as p38, JNKa and bcl-2, were detected by real-time quantitative PCR. As shown in Figure 4, although the expression of p38 appeared to be increased at 5 h and 48 h after exposure to 50 μg/kg of MC-LR, the increase was not statistically significant. Expression levels of JNKa at 5 h and bcl-2 at 24 h and 48 h after exposure to 50 μg/kg of MC-LR were significantly increased (*p* < 0.05). In the group treated with 120 μg/kg of MC-LR, the expression of p38 was significantly increased at 5 h (a 5.99 fold increase) and 48 h compared to the control group (*p* < 0.05). The expression trends of JNKa in the 50 μg/kg MC-LR treated group changed in a way that was parallel to that of the 120 μg/kg MC-LR treated group. The highest expression (6.23 fold increase) was found 5 h after exposure to 120 μg/kg of MC-LR, compared to the control group (*p* < 0.05). Expression of bcl-2 was found to have significantly increased at 24 h and 48 h, compared to the control group (*p* < 0.05). A 9.38 fold increase in Bcl-2 was found after 48 h of exposure to 120 μg/kg MC-LR. 

### Effects of MC-LR on hepatocellular apoptosis

**Figure 5:**
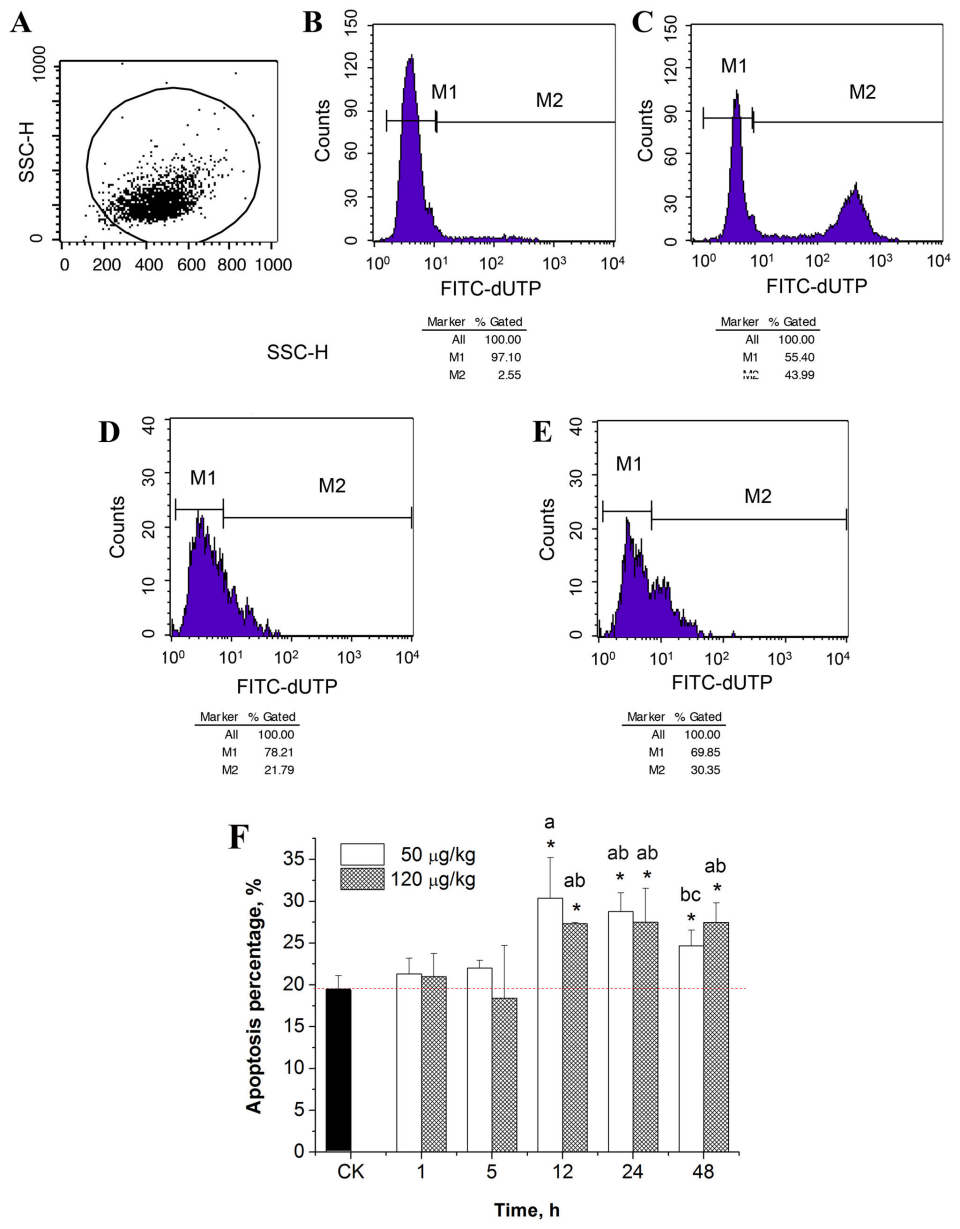
Flow cytometry analysis of liver cell apoptosis. (A) Flow cytometry analysis of the selected cells; (B) negative control from the TUNEL kit; (C) positive control from the TUNEL kit; (D) cell apoptosis in the control group; (E) 50 μg/kg MC-LR treated group at 12 h; (F) Percentage of apoptotic cells, n = 4. Asterisk indicates the statistical difference relative to the control. M1 represents normal cells *vs*. the total number of cells, M2 represents the apoptotic cells *vs*. the total number of cells. Duncan’s test was used to determine the significant difference between groups. The same letter indicates no significant difference between groups, while different letters indicate a significant difference between groups with *p* < 0.05.

The TUNEL stained hepatic cells suspension was analyzed by flow cytometry and the percentage of apoptotic cells was calculated (Figure 5). As shown in Figure 5F, a significant increase in apoptotic cells was found at 12- 48 h in different MC-LR treated groups (*p* < 0.05). The highest percentage of apoptotic cells was found at 12 h in the 50 μg/kg treated group (*p* < 0.05), which was about 156.6 percent of the control group. Moreover, with further prolongation of the exposure time, the percentage of apoptotic cells in the 50 μg/kg treated group tended to decline and a significant decrease appeared at 48 h, compared to that at 12 h (*p* < 0.05). This phenomenon was, to some extent, supported by direct immunofluorescence and confocal microscopic studies, in which the evident hollow nuclei with condensed chromatin and the exhibited apoptotic properties were observed at 12 h (Figure 3M).

## Discussion

As a liver-specific toxin, MC-LR is a potent inhibitor of protein phosphatase 1 (PP1) and protein phosphatase 2A (PP2A). In addition, previous studies have suggested that MC-LR might be able to induce excessive production of ROS [21]. The association between the inhibition of protein phosphatases and the over-production of ROS is still controversial and under investigation [13]. In this study, we use carp as a model to study crosstalk between these two potential mechanisms of MC-LR toxicity.

Many natural or synthetic compounds can induce excessive production of ROS, such as O_2_^•-^, •OH and H_2_O_2_ [13,22]. Oxidative stress that is closely related to over production of ROS is an indicator of interference with the body's normal redox state. Hydroxyl radicals, the most harmful free radicals in the body, can cause lipid peroxidation, protein peroxidation and DNA oxidative damage by attacking proteins, unsaturated fatty acids, DNA and other macromolecules. 

It has been shown in many studies that MCs can cause oxidative stress both *in vitro* and *in vivo* [3, 23-27]. However, the levels of free radicals *in vivo* have never been determined, due to technical limitations involved in the measurement of these free radicals. As mentioned above, however, EPR has proven to be the most reliable method for measuring free radicals. In this study, we provide direct evidence of the production of hydroxyl radicals induced by MC-LR in carp liver, using the EPR technique. The hyperfine parameters obtained through computer-assisted fitting analyses are consistent with previous reports in which PBN captured •OH to generate PBN-•OH adducts [28,29]. Thus, we believe the reactive oxygen species induced by MC-LR in carp liver is •OH. In addition, we observed that the •OH levels in the fish liver significantly increase during the early stage of MC-LR stress. In particular, the •OH level was significantly increased in the group treated with 120 μg/kg, one hour post MC-LR injection.

ROS production in animal cells is mainly associated with the mitochondrial metabolism. Ding et al. [30] observed a surge of the mitochondrial Ca^2+^ level in rat hepatocytes after a 10 min exposure to 1 μM MC-LR, resulting in the subsequent onset of membrane permeabilization transition (MPT). This led to the production of ROS, loss of the mitochondrial membrane, MPT, and the release of apoptotic factors, including cytochrome c, triggering apoptosis [21]. ROS plays a critical role by serving as the second messenger in the MC-LR toxicity pathway. In addition, it has also been proven that MC-LR induces the production of ROS in the leaves of aquatic plants, leading to lipid peroxidation and resulting in ultrastructural damage [31]. In the present study, we observed that MC-LR induced the generation of ROS in carp liver cells, which mediated oxidative damage, apoptosis and necrosis, as well as the destruction of the cytoskeleton. HSP70, a biomarker that is widely used to evaluate environmental stress in aquatic organisms, can protect the body from oxidative stress and apoptosis [32]. Liver HSP70 induction may rely on the perturbation of the cellular redox status. In our previous study, the expression of HSP70 was dramatically increased following exposure to dissolved MC-LR at 1.0 to 10.0 μg/L [17], which indicates its important role as a molecular chaperone under oxidative stress and explains the high tolerance of *C. carpio* to dissolved MC-LR under common environmental concentrations. In the present study, a significant increase of the expression of HSP70 was also observed in the 50 μg/kg group after 12 h and 24 h exposures to MC-LR and in the 120 μg/kg group after 24 h and 48 h exposures to MC-LR. To some extent, this over-expression of HSP70 in fish liver might contribute to alleviate MC-LR toxicity, which could manifest itself by the reduction in symptoms of hepatic cytoskeletal disruption and apoptosis in the later stage of the trial, particularly apparent in the 50 μg/kg treated group. However, the expression of HSP70 cannot reverse the damage caused by MC-LR to liver tissue.

MC-LR stress causes inhibition of protein phosphatases, resulting in over-phosphorylation of many proteins, which can further lead to rearrangement of the cell’s intermediate filament network and destruction of the cytoskeleton [33]. Beta-tubulin, one of the major components of the cytoskeleton and an indicator of cytoskeletal damage, may be associated with oxidative stress. We observed that a noticeable reduction in the number of microtubules in liver cells and the appearance of densely aggregated microtubules retracted around the nucleus occurred consistently with a high level of •OH induced by MC-LR. In addition, NAC, a GSH precursor and a commonly used anti-oxidant, can protect the cytoskeleton of carp liver cells. On the contrary, BSO, which is specific to GSH synthesis, increased the amount of damage to the cytoskeleton. In addition, GSH is a major cellular nonprotein thiol reductant and participates in numerous cellular processes, such as intermediary metabolism and protection of cells against oxidative stress [14,17]. Indeed, a noticeable depletion of intracellular GSH could be observed in the liver of fish exposed to 50 μg/kg and 120 μg/kg of MC-LR at 5 h – 12 h (results not shown), which could, in turn, alter the intracellular redox status. The decreased GSH level and the over-production of ROS could disturb the assembly of microtubules and destroy their stability [14]. As mentioned above, the high level of MC-LR could induce ROS formation in fish hepatocytes within a relatively short exposure time (1 h). Therefore, we believe MC-LR-induced ROS formation may play an important role in the disruption of microtubule structure, as observed in the present study. 

MC-LR-induced apoptosis in mammalian cells has been widely confirmed. Gehringer [34] reported that MC-LR could cause dual cellular effects (dualistic response). This means that at a low-dose (≤ 20 μg/kg *in vivo*) MC-LR successfully induced cell proliferation, while high doses (≥ 32 μg/kg *in vivo*) resulted in apoptosis/necrosis. In our present study, apoptosis was detected in MC-LR treated carp using immunohistochemistry and flow cytometry. We found that both low and high doses of MC-LR could induce significant apoptosis after a 12 h exposure. However, low dose effects on apoptosis were more obvious, as high doses of MC-LR induced necrosis. Multiple genes regulate apoptosis, such as the bcl-2 and the caspase families, as well as oncogenes like c-myc, and tumor suppressor genes like p53. Mitogen-activated protein kinase (MAPK) is an important eukaryotic signal transduction pathway and plays a key role in the regulation of gene expression and cytoplasmic activities. c-Jun amino-terminal kinase (JNK) and p38 play an important role in stress reactions, such as inflammation and apoptosis. The expression levels of JNK and p38 were significantly increased after exposure to low and high doses of MC-LR, indicating ROS mediated cell apoptosis. Oxidative stress has been shown to induce JNK activation, phosphorylation of bcl-2 and Bcl-xL, as well as to promote apoptosis [21,35]. The JNK and p38 pathways usually present synergistic apoptotic signals, which we also found to be the case in our present study. Overexpression of bcl-2 was found in both low and high dose MC-LR treated groups. Bcl-2 is an anti-apoptotic gene and its overexpression is assumed to reduce production of oxygen free radicals and lipid peroxidation. Moreover, overexpression of bcl-2 can also increase production of GSH and other antioxidants. Although the over-expression of bcl-2 enhanced resistance to some degree, the damage to fish liver caused by MC-LR could not be reversed. 

In our present study, a significant induction of hydroxyl radicals (•OH) was observed in carp liver after exposure to MC-LR. This provides evidence for oxidative stress as the toxic mechanism induced by MC-LR. Excessive production of ROS and inhibition of protein phosphatase triggers a series of pathological effects, including destruction of the cytoskeleton. Pre-injection of the antioxidant NAC has a significant protective effect on the carp liver cytoskeleton. On the contrary, BSO exacerbates the damage to the cytoskeleton. ROS could induce the expression of apoptosis-related genes, including p38 and JNKa. A significant increase in apoptotic cells was observed 12 - 48 hours post-exposure. Apoptosis was reduced after 48 h, which may be related to the upregulation of bcl-2 and the previous over-expression of HSP70. Our study further supports the role of ROS in MC-LR induced liver damage in carp, and provides a basis for the ongoing study of the molecular mechanisms behind MC-LR toxicity. 
